# Preparation of CdS@C Photocatalyst Using Phytoaccumulation Cd Recycled From Contaminated Wastewater

**DOI:** 10.3389/fchem.2021.717210

**Published:** 2021-09-29

**Authors:** Jia-Xin Li, Rou-Lan Zhang, Zi-Jian Pan, Yan Liao, Chao-Bin Xiong, Ming-Li Chen, Rong Huang, Xiao-Hong Pan, Zhi Chen

**Affiliations:** ^1^ Fujian Provincial Key Laboratory of Soil Environmental Health and Regulation, College of Resources and Environment, Fujian Agriculture and Forestry University, Fuzhou, China; ^2^ State Key Laboratory of Ecological Pest Control for Fujian and Taiwan Crops and Key Lab of Biopesticide and Chemical Biology, Ministry of Education, College of Plant Protection, Fujian Agriculture and Forestry University, Fuzhou, China

**Keywords:** *Pistia stratiotes*, cadmium sulfide, photocatalyst, phytoaccumulation, degradation

## Abstract

Cadmium is one of the most toxic heavy metal contaminants in soils and water bodies and poses a serious threat to ecosystems and humans. However, cadmium is also an important resource widely used in many industries. The recovery of cadmium in the form of high-value products is considered as an ideal disposal strategy for Cd-contaminated environments. In this work, *Pistia stratiotes* was used to recycle cadmium from wastewaters through phytoaccumulation and then transformed into carbon-supported cadmium sulfide photocatalyst (CdS@C) through carbonization and hydrothermal reaction. The CdS@C photocatalyst contained a mixture of cubic and hexagonal CdS with lower band gap energy (2.14 eV) and high electron-hole separation efficiency, suggesting an excellent photoresponse ability and photocatalytic efficiency. The impressive stability and photocatalytic performance of CdS@C were demonstrated in efficient photodegradation of organic pollutants. •OH and O_2_•- were confirmed as the major active species for organic pollutants degradation during CdS@C photocatalysis. This work provides new insights into addressing Cd contaminated water bodies and upcycling in the form of photocatalyst.

## Introduction

Cadmium is one of the most important heavy metal contaminants in soils and water bodies and is extremely toxic to plants, animals, and humans (Zhao et al., 2015; Gong et al., 2019; Xing et al., 2020; Zhou et al., 2020). Therefore, the remediation of Cd-contaminated aqueous and soil environments has become a matter of great concern in recent years. But on the other hand, cadmium is also an essential resource, which was widely used in many industries, including electroplating, photovoltaics, and metal smelting (Celik et al., 2017; Kruglikov et al., 2019; Zhong et al., 2021). Although there are a number of options for limiting the transfer of Cd in the environments and accumulating in the food chain, the ideal remediation strategy for Cd-contaminated environments is the recovery or recycling in the form of high-valued products (Chen et al., 2020).

Phytoextraction is considered as a feasible and cost-efficient *in situ* remediation strategy for slightly or moderately contaminated environments with heavy metals, including Cu, Mn, As, Cd, and Zn (Hou et al., 2017; Li et al., 2018; Ent et al., 2020). In fact, phytoextraction has been applied for achieving a substantial removal of arsenic (As) and cadmium (Cd) from environments at large-scale field trials in China (Li et al., 2018; Zhou et al., 2020). In past decades, the majority of present works have focused on enhancing the removal capacity of heavy metals from environments using hyperaccumulator plants. However, fewer studies have attempted to find an appropriate method for recovery and recycling of heavy metal resources after phytoextraction, except for noble metals, such as Ni, Au, Cu, and Pt (Guilpain et al., 2018; Doroshenko et al., 2019; De Bernardi et al., 2020). As non-noble metals, Cd is deemed to be not valuable and economically viable for recycling, and most of the Cd-accumulated plants were disposed of in a landfill as hazardous waste (Cui et al., 2018).

More recently, some metal-loaded biomass has been recycled in the form of new functional biochar materials, and advanced as great potential of environmental applications (Harumain et al., 2017; Chen et al., 2019; Chen et al., 2020; Li et al., 2019a). For example, Cu accumulated cotton leaves could be recovered into Cu nanoparticle-embedded biochar with high performance of cyanobacteria inhibition through pyrolysis (Li et al., 2019a). Harumain and co-workers suggested that palladium containing plant biomass could be converted into carbon-supported nano-catalysts by the treatment of pyrolysis (Harumain et al., 2017). Besides, our previous works have suggested that Cd-enriched hyperaccumulator biomass could be recycled in the form of CdS@C nanocomposite through pyrolysis carbonization and hydrothermal reaction and acted as catalyst in dyeing wastewater treatment by photodegradation (Chen et al., 2019; Chen et al., 2020). Compared with pure CdS, the as-prepared CdS@C nanocomposite exhibited considerably higher light-harvesting capacity and photocatalytic efficiency due to low band gap energy and effective electron-hole separation (Ma et al., 2017; Zhu et al., 2017; Bantawal et al., 2019; Cao et al., 2019; Shi et al., 2019; Chen et al., 2020).

In light of these knowledges, we proposed a strategy for upcycling of Cd into high-performance carbon-supported CdS nanocomposite photocatalyst extraction from slightly or moderately contaminated wastwaters using hydrophyte. In this work, *Pistia stratiotes* was used to adsorb and accumulate Cd from slightly or moderately contaminated wastwaters, and then recovered in the form of CdS@C photocatalyst. The visible light–driven photocatalytic performance and mechanism of CdS@C in the organic pollutant degradation were investigated.

## Experimental Section

Materials: Cadmium chloride (CdCl_2_.9H_2_O, 98%), sodium sulfifide (Na_2_S·2.5H_2_O, ≥ 98%), rhodamine B (RhB, 95%), terephthalic acid (TA, 99%), and 5,5’–dimethyl–1–pyrroline–N–oxide (DMPO, 97%) were commercially purchased. *Pistia stratiotes* was collected from Fujian Agriculture and Forestry University, Fujian Province, China.

Cd treatment experiments: The seedling of *Pistia stratiotes* were grown in plastic pots with nutrient solutions containing a quarter of Murashige and Skoog (MS) salts at 25°C (16 h light cycle) for 2 weeks, and then transferred to Cd treatment experiments. For Cd treatment experiments, plants were grown in a quarter of MS solution containing different concentrations of Cd ranging from 5 to 15 mg/L.

**SCHEME 1:**
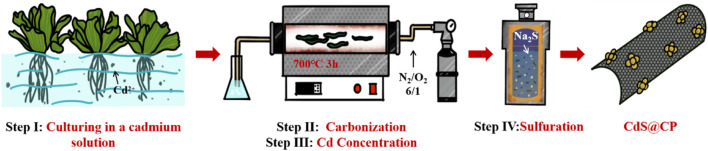
Schematic illustration of the procedures for CdS@CP photocatalyst composites.

Preparation of CdS and CdS@CP photocatalyst composites: After Cd accumulation, the Cd-enriched *Pistia stratiotes* was converted into the CdS@C photocatalyst as shown in Scheme 1. Briefly, the roots and leaves of plant cultivation with Cd solution were washed, dried, and grounded into powder. The samples were then carbonization at 700 °C for 3 h under nitrogen atmosphere in a tubular furnace to obtain *Pistia stratiotes*–derived biochar (CP). The Cd-containing biochar were further carbonized at 650°C for 1 h under the mixed atmosphere of N_2_/O_2_ (6/1, v/v) to increase the content of Cd to obtain Cd-enriched CP (Cd-CP). After washing and drying, the samples were mixed with 5 mM Na_2_S solution and reacted at 180°C for 72 h in Teflon-lined stainless steel vessel to obtain CdS@CP catalyst.

The pure CdS was prepared as reported by previous work (Huang et al., 2018). Briefly, Cd(CH_3_COO)_2_·2H_2_O was dissolved in ethanediamine and mixed with thioacetamide through stirring for 1 h. The mixture was then reacted in sealed stainless steel vessel with Teflon-lined at 180°C for 5 h. The as-prepared CdS was obtained by filtration, washing, and drying.

Characterization: The contents of Cd, C, H, and S in the material were measured by elemental analyzer (EA) and inductively coupled plasma mass spectrometry (ICP-MS). The surface morphology of the CdS@CP photocatalytic synthesis was observed by JSM6700-F scanning electron microscope (SEM) at 10 kV. Using Escalab 250 X-ray photoelectron spectroscopy (XPS), the states of Cd, S, and C elements in the composites were analyzed at 0.6 eV with Al/Mg double anode target as radiation source. The phase of the material was analyzed by Ultima IV X-ray powder diffraction (XRD). The crystal structure of the composite was observed by JEM-2010 transmission electron microscope (TEM). Using a Shimazu UV-2550 UV-Vis spectrometer, the composite band gap width was calculated by the K-M formula. Kubelka–Munk (K-M) formula: d (*Ahv/K*)^2^ = *h*_*v*_ *-E*_*g*_, where A is the absorbance, h_v_ is the 1240/wavelength, K is the constant, and E_g_ is the band gap width. Hydroxyl radical (•OH) was detected by means of fluorescence measurements in the presence of terephthalic acid (TA). Superoxide radical (O_2_•-) were detected by performing EPR spin trapping in the presence of DMPO.

Photocatalytic reaction: 50 ml of 40 mg/L RHB dye solution was mixed with 20 mg of CdS@CP photocatalytic composite material in the dark at 700 rpm on a magnetic agitator for 30 min. Then CEL-HXF300 xenon lamp was used for illumination photocatalysis for 60 min, and samples of the mixed solution were collected every 10 min. The sample was recorded by a 554 nm ultraviolet-visible spectrophotometer. The degradation efficiency was calculated by the following formula:

R=Ct/C0×100%,

where R is the degradation rate, C_t_ is the absorbance of RhB measured at each sampling, and C_0_ is the initial absorbance of RhB without catalytic reaction.

Photocatalyst mechanism explore: The electrochemical workstation (Shanghai Chenhua CHI600E) was used to evaluate the photocurrent generated during the photocatalytic process of the catalyst. In brief, the samples were dispersed in naphthene solution (5%) and dropped onto indium tin oxide (ITO) glass substrates (1 cm × 1 cm). The photocurrent was measured with zero bias in 0.2 M Na_2_SO_4_ aqueous solution using the sample-coated ITO. The transient photocurrent was recorded through electrochemical workstation with switch on and -off visible irradiation in 30-s intervals. An electron paramagnetic resonance (EPR) spectrometer was used to determine the superoxide radical (O_2_•^-^) generated by the excitation of the trapping agent DMPO and the photocatalyst in the methanol solution under light irradiation. Hydroxyl radical (•OH) was detected by means of fluorescence measurements in the presence of terephthalic acid (TA).

## Results and Discussion

### Preparation and Characterization of CdS@C Photocatalyst

In this work, the Cd adsorption capacity of *Pistia stratiotes* was investigated at different initial concentrations ranging from 0 to 15%. As shown in Supplementary Figure S1, it was found that *Pistia stratiotes* had excellent adsorption ability on different concentrations of Cd. The amount of Cd in roots and leaves of *Pistia stratiotes* were further analyzed as shown in Supplementary Table S1. The results indicated that the Cd adsorption amounts of roots and leaves were increased from 75 mg/kg to 1089 mg/kg with the increase of initial Cd concentration from 5 mg/L to 15 mg/L. And the roots of *Pistia stratiotes* had the maximum Cd enrichment capacity (1089 mg/kg) at initial Cd concentration of 15 mg/L in solution. Thus, the roots of *Pistia stratiotes* was further used for preparation of CdS@C photocatalyst through carbonization and hydrothermal sulfuration process.

**FIGURE 1:**
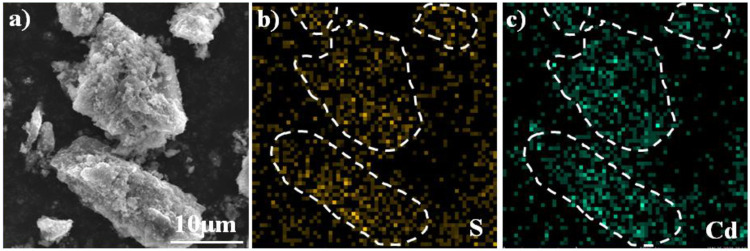
SEM images of **(A)** CdS@CP, surface energy spectrum scan of **(B)** S and **(C)** Cd.

In order to increase the CdS content of synthetic material with high photocatalytic performance, Cd was further concentrated using secondary pyrolysis of Cd-containing biochar under a N_2_/O_2_ atmosphere at 650°C for 1 h after anaerobic carbonization at 700°C. Elemental analysis indicated that the content of C and Cd was ca. 37.49 and 5.93% in the synthetic materials (Supplementary Table S2). The BET surface area of the CdS@CP was about 136 m^2^ g^−1^, which was lower than other biochar materials (Huang et al., 2018). In the process of hydrothermal vulcanization, the addition of sodium sulfide leads to the increase of S content in the composite (5.02%). Scanning electron microscope (SEM) was used to observe the surface morphology and microstructure of carbon-based and synthetic materials. Obvious microporous structure and unknown particles can be observed on the surface of CdS@CP. Through the energy spectrum scan of the selected area, it is found that these particles are mainly composed of Cd and S elements (Figure 1). These results indicate that the prepared composite material contains a large amount of sulfur and cadmium and may be supported on the carbon-based surface in the form of CdS.

**FIGURE 2:**
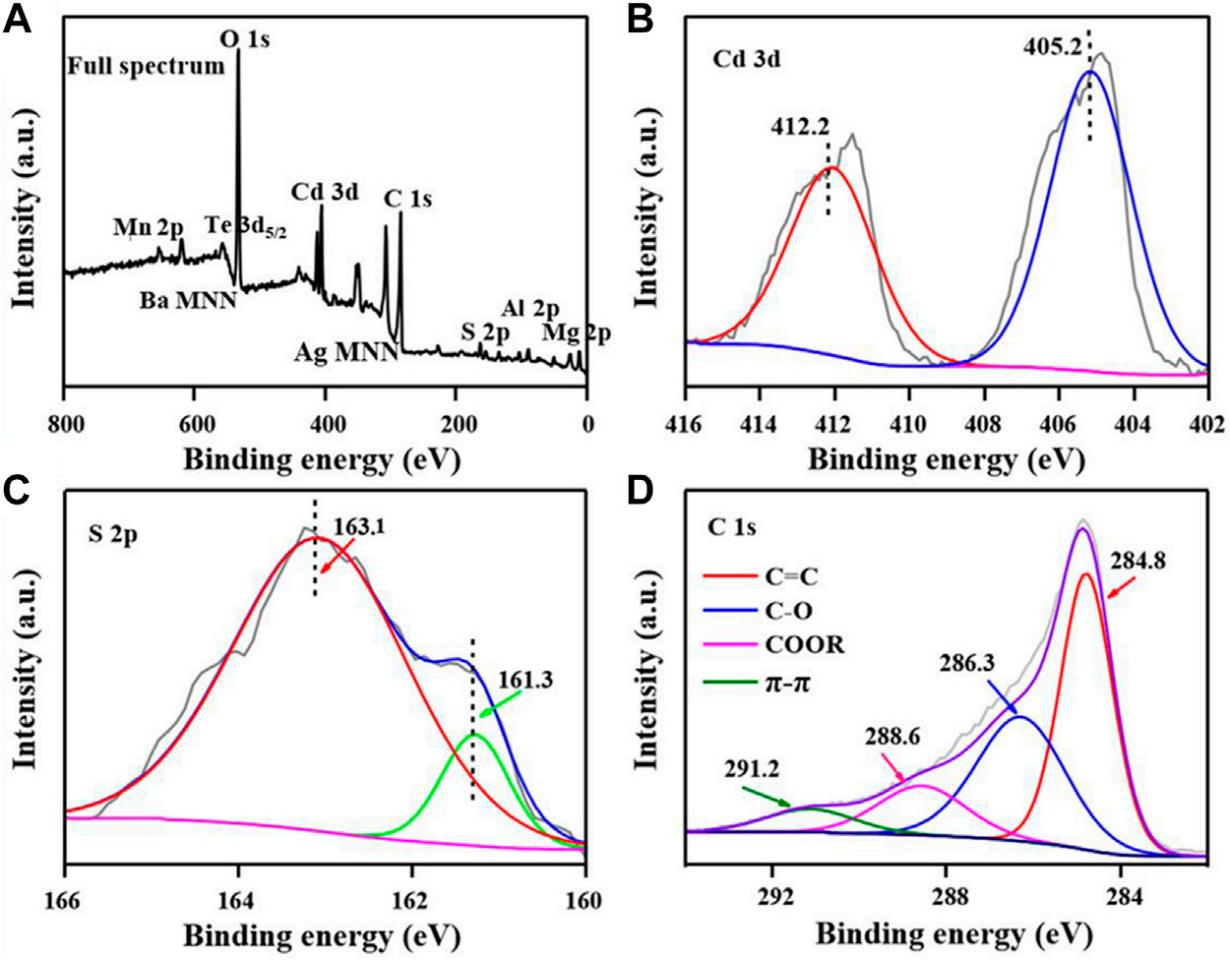
XPS spectra of CdS@CP photocatalyst. **(A)** Full spectrum; **(B)** Cd 3d spectrum; **(C)** S 2p spectrum; **(D)** C 1s spectrum.

Furthermore, XPS was used to characterize the chemical composition, element valence, and surface functional groups of CdS@CP. According to the XPS spectrum, strong signal peaks of O 1s, Cd 3d, C 1s, and S 2p existed in the composite samples, while the miscellaneous peaks suggested the present of other mineral elements, such as Mn, Ba, Ag, Al, and Mg (Figure 2). The binding energy of Cd is shown in Figure 2B. The convolution peaks of 412.2 and 405.2 eV correspond to Cd 3d5/2 and Cd 3d5/2, which indicated the characteristic peaks of the Cd^2+^ species, whereas the two single peaks of 163.1 eV (S 2p1/2) and 161.3 eV (S 2p3/2) were considered the characteristic peaks of the S^2-^ (Li et al., 2019b; Fan et al., 2016; Zhu et al., 2019). The binding energy of Cd 3d and S 2p indicates that the chemical composition of Cd exists in the composite. The four convolution peaks in the C 1s spectrum (Figure 2D) correspond to π-π (291.2 eV), COOR (288.6 eV), C-O (286.3 eV), and C=C (284.8 eV), respectively (Lei et al., 2017; Huang et al., 2018). These results indicate that the graphitization characteristics of biochar with oxygen-containing groups. Besides, the convolution peaks of O 1s demonstrated that the biochar support surface of CdS@CP was functionalized with–OH groups (Supplementary Figure S2).

**FIGURE 3:**
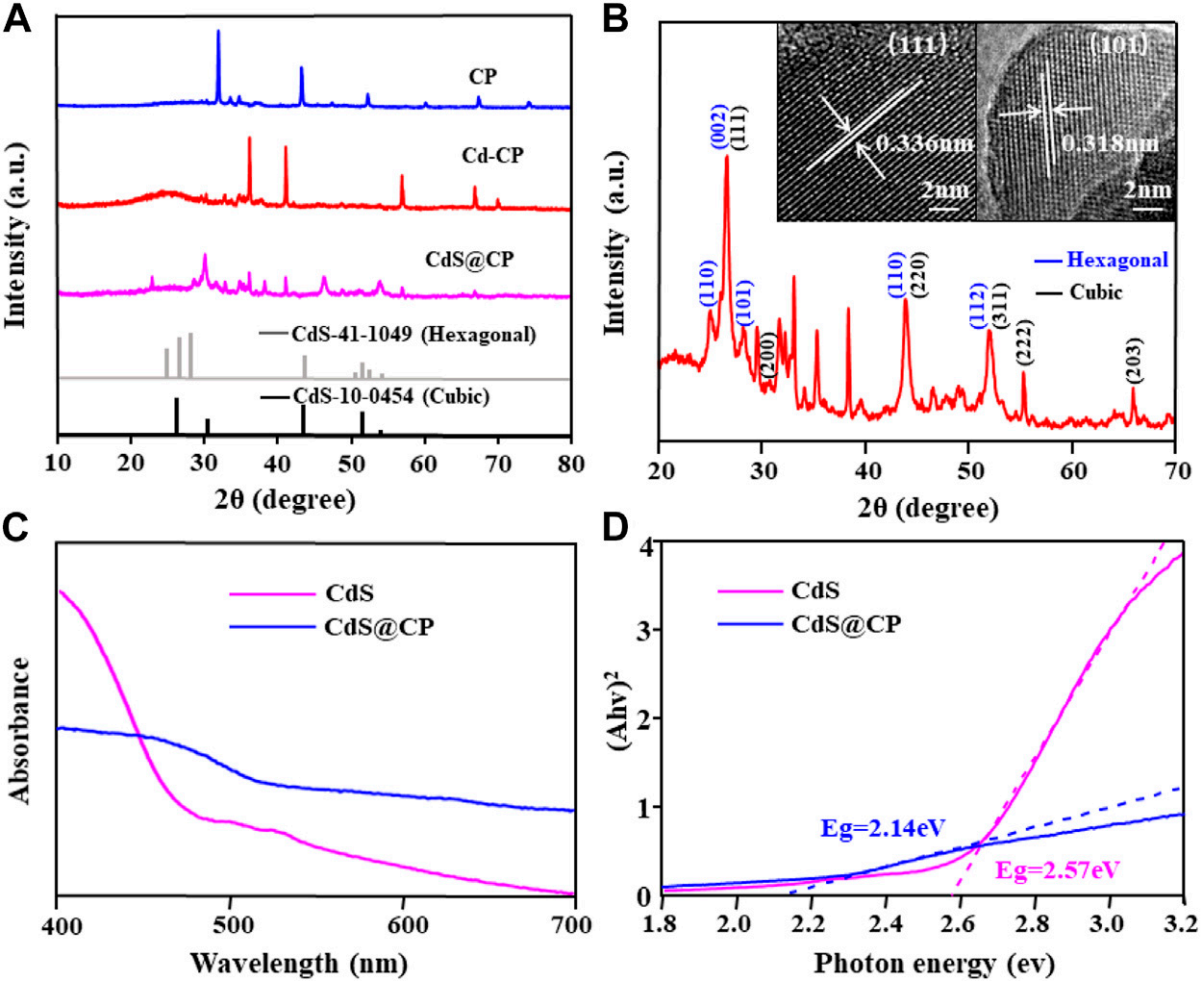
**(A)** XRD spectra of CP, Cd-CP, and CdS@CP; **(B)** HRTEM images of CDS@CP; **(C)** DRS spectra of CdS and CdS@CP; **(D)** the band-gap energy of CdS and CdS@CP.

The phase structure and crystal morphology of CdS@CP photocatalyst were observed by XRD and HRTEM. The results are shown in Figure 3, the XRD patterns of the CP, Cd-CP, and CdS@CP showed different characteristics. Therein, the diffraction peaks observed in the CdS@CP was assigned as the mixed-phase of hexagonal and cubic CdS (PDF 41–1049 and 10–0454) (Figure 3A). The HRTEM image showed crystal spacings of 0.336 and 0.318 nm on the CdS@CP, which were ascribed to the [111] and [101] crystal planes of the hexagonal and cubic CdS (Figure 3B). Therefore, the characterization by ICP, SEM, XPS, XRD, TEM, and XPS certified that Cd-enriched *Pistia stratiotes* rots were successfully converted into the CdS@C photocatalyst.

The band gap is an important indicator to evaluate the visible light absorption performance and photocatalytic activity of CdS@C composites. As shown in Figure 3C, compared with CdS, the visible light absorption capacity of CdS@CP photocatalyst increased from 450 to 520 nm, indicating that CdS@CP photocatalyst have better light collection capacity under visible light irradiation. The band gap energy of the CdS@CP (2.14 eV) was further calculated by the Kubelka–Munk formula, which was significantly lower than that of single-CdS (2.57 eV) (Figure 3D), suggesting a higher photocatalytic efficiency under visible light irradiation (Zhu et al., 2019).

### Photocatalytic performance of CdS@C in the degradation of RhB

**FIGURE 4:**
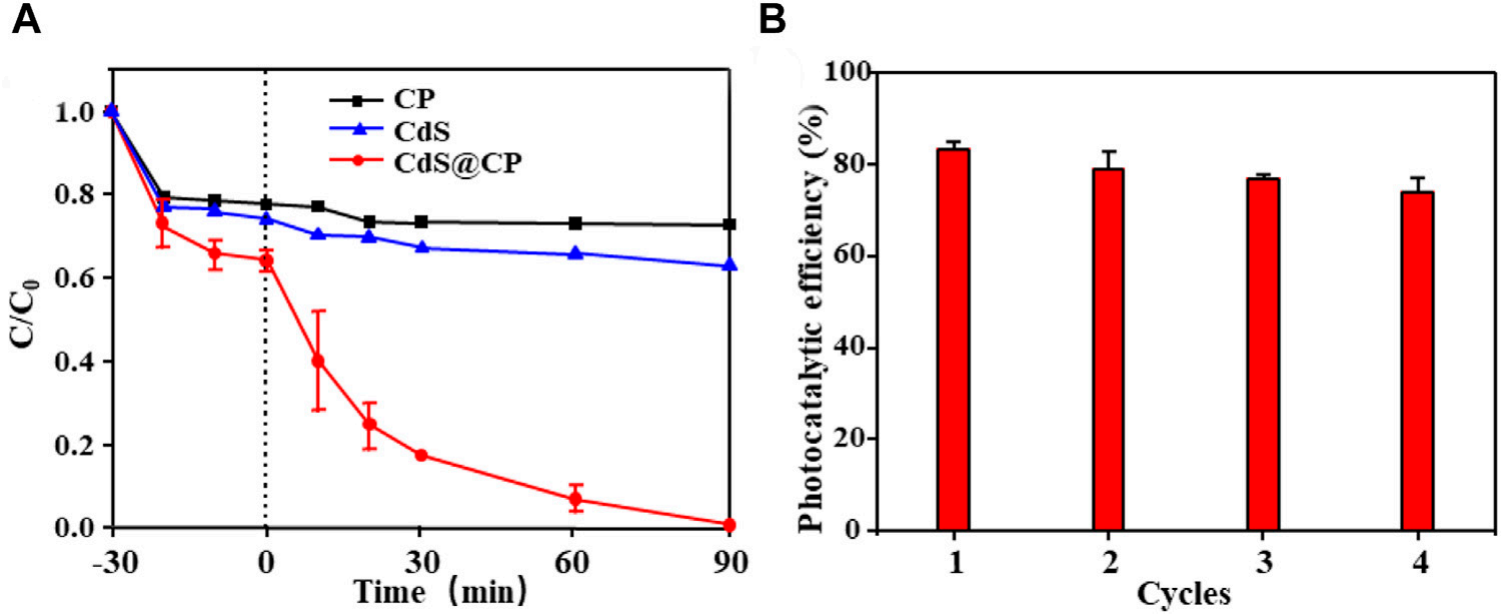
**(A)** RhB degradation eﬃciency of CP, CdS, and CdS@CP; **(B)** recycling reactions when using CdS@CP.

The photocatalytic degradation experiment of RhB dye solution was carried out by CdS@CP photocatalyst. The results are shown in Figure 4A. In the experiment, the CP and single-CdS reached the adsorption saturation point at 10 min, while the photocatalyst reached the adsorption saturation point at 20 min, which indicated that the adsorption capacity of the composite material was larger. After visible irradiation for 90 min in degradation experiment, the RhB degradation rate of single-CdS was 32% and the RhB degradation rate of CdS@CP photocatalyst was close to 99%, significantly higher than single-CdS degradation efficiency. The recycling reaction of RhB photodegradation revealed that the photocatalytic efficiency of CdS@CP could maintain more than 75% of the initial value after four consecutive treatments, indicating that the photocatalyst has stable photocatalytic performance and reusability (Figure 4B). More importantly, it was found that the release of Cd^2+^ increased rapidly to about 10 mg/L during RhB photodegradation of pure CdS, while an extremely low concentration of Cd^2+^ (about 0.1 mg/L) could be detected during RhB photodegradation of CdS@CP, suggesting a good safety and stability of CdS@CP (Supplementary Figure S3). Besides, it was confirmed that bispyribac-sodium also could be degraded effectively by photocatalytic reaction of CdS@CP (Supplementary Figure S4), suggested that CdS@CP was as an excellent photocatalyst for photodegradation of organic pollutants.

### The Catalytic Mechanism of CdS@CP Photocatalytic Composites

**FIGURE 5:**
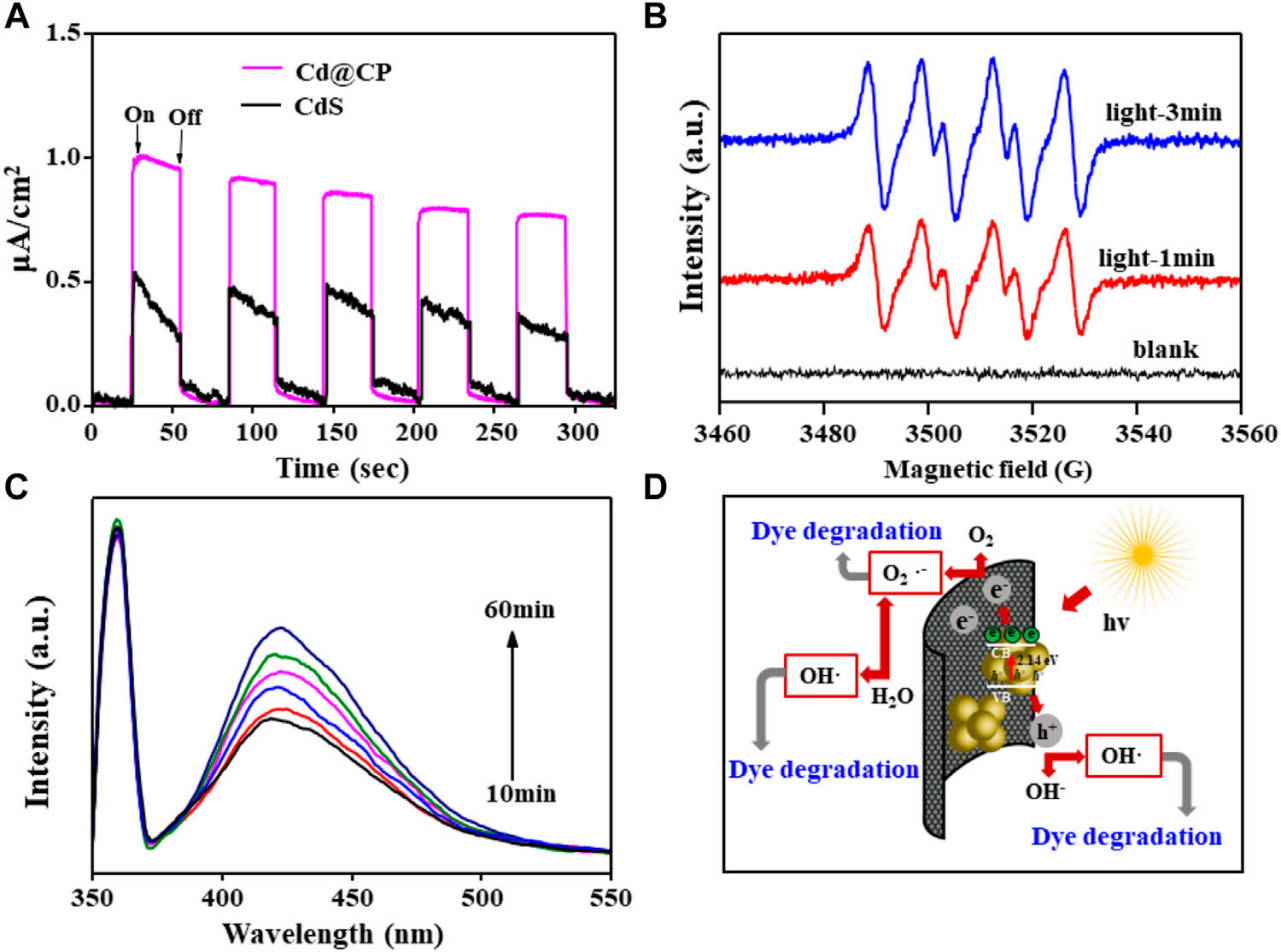
**(A)** Transient photocurrent plots for single-CdS and CdS@CP; **(B)** EPR spectrum of superoxide by DMPO for CdS@CP; **(C)** fluorescence spectra of hydroxyl radicals trapped by TAOH for CdS@CP; **(D)** photocatalytic degradation mechanism of CdS@CP.

It has been proposed that the carbonaceous carrier of CdS@C photocatalyst acts as a strong electron acceptor and then the photo-generated electrons and holes of CdS can be effectively separated by transferring electrons to the carbonaceous carrier, thereby improving the photocatalytic performance (Chen et al., 2015; Shi et al., 2019; Xue et al., 2021). We observed the instantaneous photocurrent changes of single CdS and CdS@CP photocatalysts. The results show that the maximum current intensity of a single CdS photocurrent is about 0.5 μA/cm^2^ and decreases with the passage of time under the same condition. The maximum photocurrent intensity of the composite material is about 1.0 μA/cm^2^, which is twice that of a single CdS and remains stable (Figure 5A). The result suggested that the CP in CdS@CP photocatalysts would reduce the recombination of charge and enhance separation and transport of electron-hole pairs to produce reactive species. In order to explore the main active molecules of the CdS@CP photocatalyst during photocatalytic degradation, we used a trapping agent to bind the active molecules and used EPR spectrum and fluorescence spectroscopy to measure. The EPR spectrum of Figure 5B shows that the photocatalyst has no spin signal in the dark. However, DMPO can capture the O_2_•^-^ signal after illumination, and the spin signal gradually increases with time. This indicates that the photocatalyst will continue to produce O_2_•^-^ under light. Fluorescence spectra showed that •OH existed in the catalytic process, and the fluorescence intensity also increased with the extension of time, indicating that •OH would also be produced in the illumination process and its output increased with the increase in illumination time (Figure 5C) (Wang et al., 2021). The results of EPR and fluorescence spectra show that the composites can produce O_2_•^-^ and •OH in the process of photocatalytic degradation, and these free radicals have strong oxidation properties, which may be the main factor leading to the decomposition of organic matter. Further active free radical capture experiments suggested that the addition of the scavengers (ammonium oxalate, *tert*-butyl alcohol, and benzoquinone) could cause significant decrease in RhB photodegradation efficiency of CdS@CP. The capture of O_2_•^−^ radicals with benzoquinone could largely inhibite the photodegradation of RhB, whereas the trapping of •OH and *h* ^+^ showed less effect on the photodegradation of RhB (Supplementary Figure S5).

Based on the above experimental results, the catalytic mechanism of CdS@CP photocatalytic composite material was further constructed, as shown in Figure 5D. Under visible light irradiation, the CdS composite material will absorb energy to cause electrons to transition from the valence band (VB) to the conduction band (CB) and generate electron-hole pairs. Moreover, biochar has a strong ability to accept electrons, which extends the binding time of electrons and holes. Furthermore, the photocatalytic activity of the composite was enhanced. Photogenerated electron (e^-^) is reductive and can reduce O_2_ molecules on the surface of biochar to O_2_•^-^, which can directly participate in the degradation of dyes. In addition, O_2_•^-^ can also decompose H_2_O into •OH. The strong oxidation of hole *h* ^+^ can directly oxidize H_2_O to •OH and participate in the process of photocatalytic degradation of dyes. These active molecules with strong oxidation can well oxidize and degrade organic matter into CO_2_ and H_2_O (Chen et al., 2015; Liao et al., 2019; Xue et al., 2021).

## Conclusions

In this work, *Pistia stratiotes* was used to adsorb and accumulate Cd from slightly or moderately contaminated wastwaters. It was found that the roots of *Pistia stratiotes* have excellent absorption capacity of Cd. The Cd-enriched roots of *Pistia stratiotes* could be recovered into high performance CdS@C photocatalyst through carbonization and hydrothermal sulfuration process. The CdS@CP photocatalyst exhibits superior visible light-driven photocatalytic efficiency and stability in the process of photocatalytic degradation of RhB compared with single-CdS. Research on the underlying mechanism shows that the excellent photocatalytic performance of CdS@CP was a result of the effective light collection and the rapid separation and transport of photogenerated charge carriers due to the close contact between CdS and mesoporous carbon. In addition, the formation of catalytic free radicals (•OH and O_2_•^-^) greatly promotes the excellent photodegradation of organic pollutants by CdS@C.

## Data Availability

The original contributions presented in the study are included in the article/Supplementary Material, and further inquiries can be directed to the corresponding authors.
