# Supplementary material for: Preparation of CdS@C Photocatalyst Using Phytoaccumulation Cd Recycled From Contaminated Wastewater
Source: Front Chem. 2021 Sep 29;9:717210. doi: 10.3389/fchem.2021.717210 (PMC8512432; doi:10.3389/fchem.2021.717210)
Supplement: Supplementary file 1 [file DataSheet1.docx]

Preparation of CdS@C photocatalyst using phytoaccumulation Cd recycled from contaminated wastewater

Jiaxin Li^1^, Ruolan Zhang^1^, Zijian Pan^1^, Yan Liao^1^, Chaobin Xiong^1^, Mingli Chen^1^, Rong Huang^1^, Xiaohong Pan^2,^*, Zhi Chen^1,^*

^1^Fujian Provincial Key Laboratory of Soil Environmental Health and Regulation, College of Resources and Environment, Fujian Agriculture and Forestry University, No. 15 Shang Xia Dian Road, Fuzhou, Fujian350002, China

^2^ State Key Laboratory of Ecological Pest Control for Fujian and Taiwan Crops & Key Lab of Biopesticide and Chemical Biology, Ministry of Education, College of Plant Protection, Fujian Agriculture and Forestry University, Fuzhou, Fujian 350002, P. R. China

* Corresponding author: Dr Xiaohong Pan; Dr Zhi Chen

E-mail: panxiaohong@163.com, Tel/Fax: (+086)591-83789258 (Xiaohong Pan).

Email: chenzhi0529@163.com, Tel/Fax: (+086)591-86398509 (Zhi Chen)

*Number of pages: 2*

*Number of tables: 2*

*Number of figures: 1*

**Figure list**


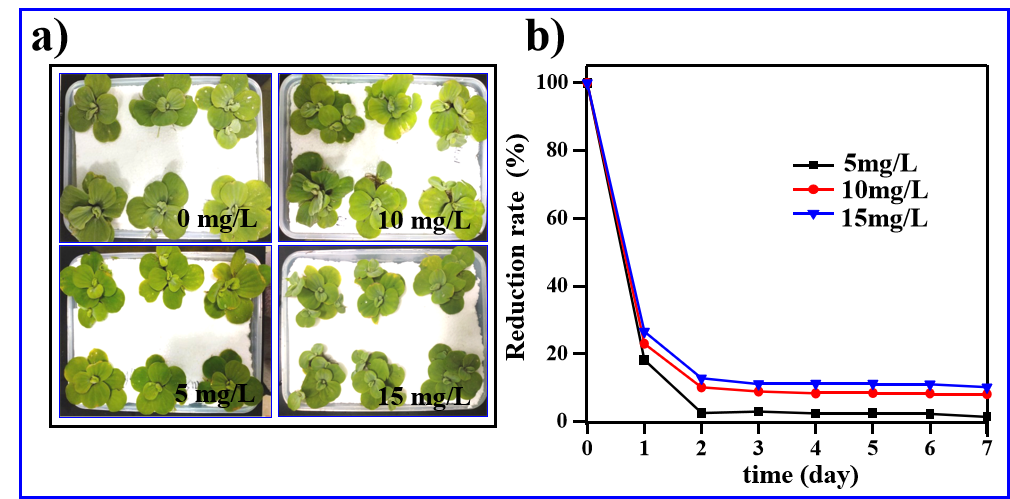


**FIGURE S1** (a) Cultivation of *Pistia stratiotes* in different Cd concentrations; (b) Cd reduction by *Pistia stratiotes*.


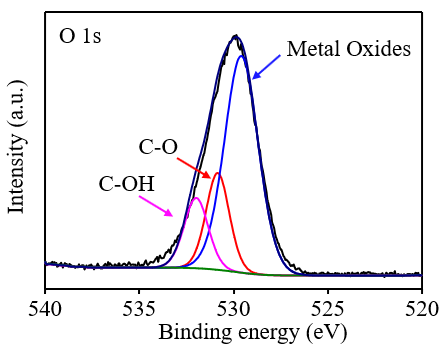


**FIGURE S2** The O 1s convolution peaks of CdS@CP XPS spectra.


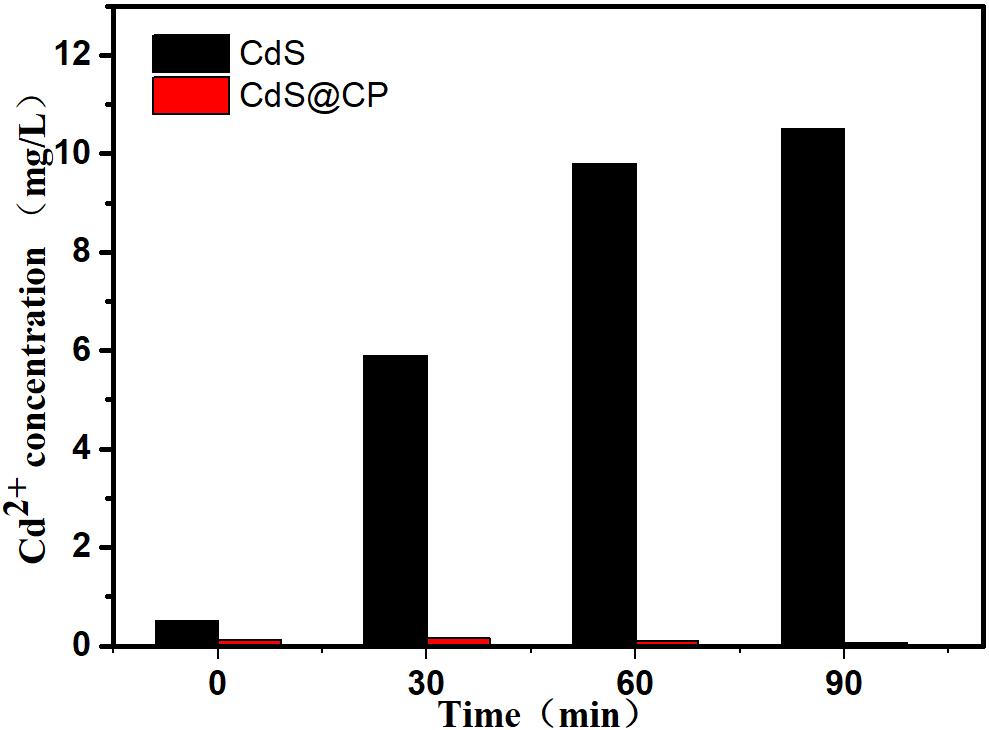


**FIGURE S3** The release of Cd^2+^ in the catalytic systems of CdS and CdS@CP


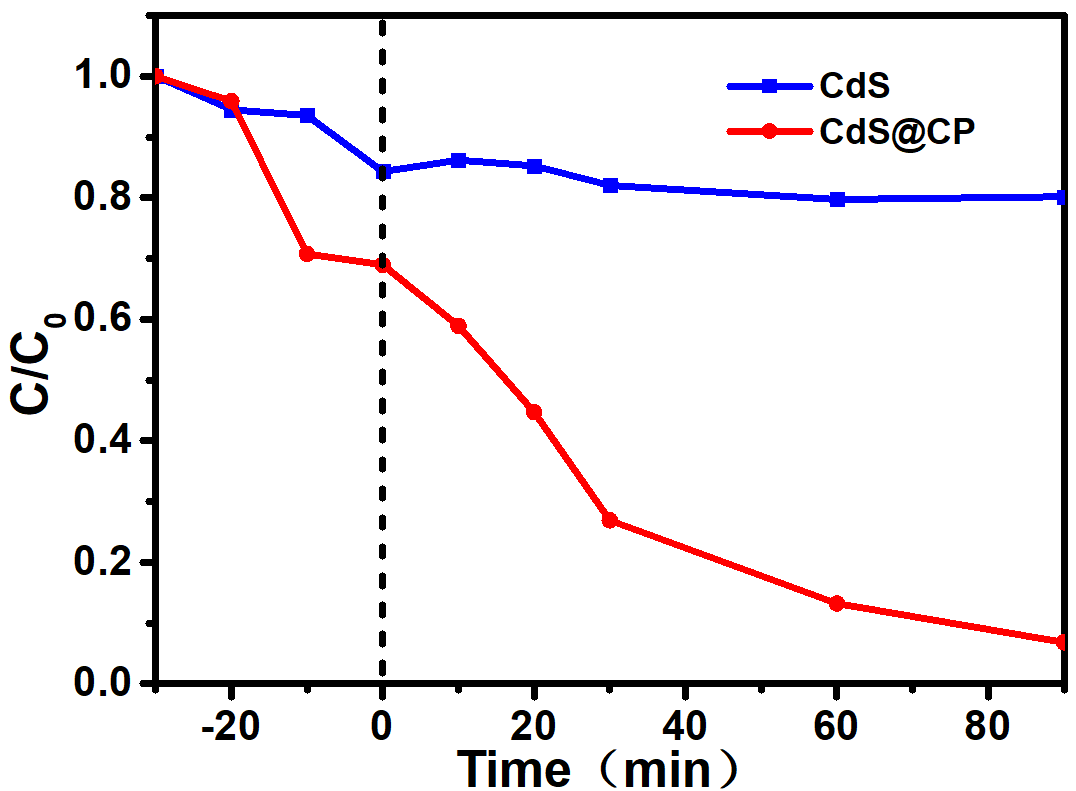


**FIGURE S4** Bispyribac-sodium photodegradation eﬃciency of CdS@CP


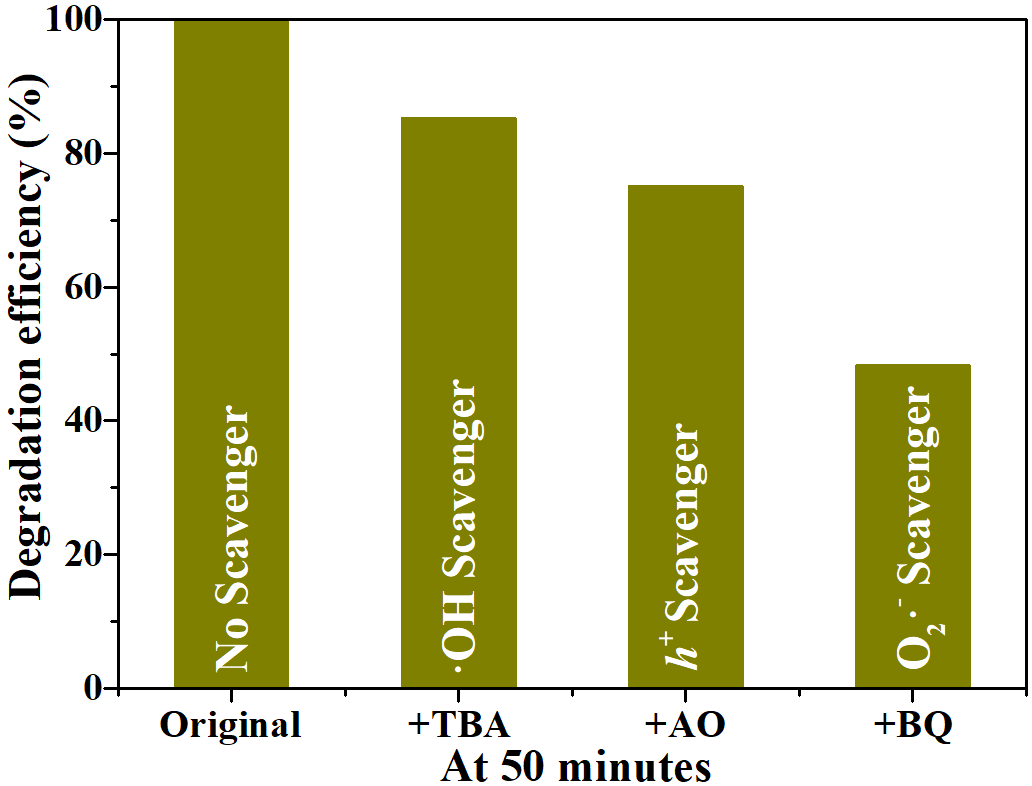


**FIGURE S5** Photocatalytic efficiency of CdS@C with exposure to various scavengers. TBA represents *tert*-butyl alcohol; AO represents ammonium oxalate; BQ represents benzoquinone.

**Table list**

**TABLE S1** The content of Cd in *Pistia stratiotes*

| Sample | Cd concentration in treatments  (mg/L) | Cd in leaf(mg/kg) | Cd in root (mg/kg) |
| --- | --- | --- | --- |
| *Pistia stratiotes* | 5 | 75±8 | 219±28 |
|  | 10 | 222±24 | 588±52 |
|  | 15 | 347±38 | 1089±63 |

**TABLE S2** Elemental content and BET surface areas of CdS@CP

| Samples | Contents（%） | | | | S_BET_ (m^2^·g^-1^) |
| --- | --- | --- | --- | --- | --- |
|  | C | H | S | Cd |  |
| CdS@CP | 37.49 | 1.62 | 5.02 | 5.93 | 136 |
